# Santé bucco-dentaire des Pygmées Baka dans la ville de Dimako au Cameroun: une étude transversale de 205 cas

**DOI:** 10.11604/pamj.2024.49.25.44222

**Published:** 2024-09-30

**Authors:** Lionel Berthold Keubou Boukeng, Charly Eboko Etoa, Leonie Dapi Nzefa, Ariane Nouko, Claude Axel Minkandi, Jean Yves Bevela, Djouwairiyatou Sali

**Affiliations:** 1 Direction de la Lutte contre la Maladie, les Epidémies et les Pandémies, Ministère de la Santé Publique, Yaoundé, Département de Santé Publique et Sciences Sociales, Faculté de Médecine et des Sciences Pharmaceutiques de Sangmélima, Université d’Ebolowa, Sangmélima, Cameroun,; 2 Département de Santé Publique, Faculté de Médecine et des Sciences Biomédicales, Université de Yaoundé I, Yaoundé, Cameroun,; 3 Faculté de Médecine et d'Optométrie, Institut de Santé et des Sciences de la Vie, Université de Linneaus, Kalmar, Suède,; 4 Délégation Régionale de la Santé Publique du Centre, Ministère de la Santé Publique, Yaoundé, Cameroun,; 5 Délégation Régionale de la Santé Publique de l'Est, Ministère de la Santé Publique, Bertoua, Cameroun,

**Keywords:** Carie dentaire, gingivite, connaissances, attitudes, pratiques, Pygmées, Baka, Dimako, Cameroun, Dental caries, gingivitis, knowledge, attitude, practice, Pygmies, Baka, Dimako, Cameroon

## Abstract

**Introduction:**

le profil épidémiologique des maladies bucco-dentaires se caractérise par des disparités entre les groupes spécifiques. L'objectif général de cette étude était d'évaluer l'état de santé bucco-dentaire des Pygmées Baka à Dimako au Cameroun.

**Méthodes:**

nous avons mené une étude transversale entre le 1^er^ janvier et le 14 juin 2021 dans les campements Baka à Dimako. Au total, 205 personnes âgés d'au moins 12 ans ont été recrutés avec un échantillonnage non probabiliste, de type consécutif. Les données ont été collectées avec un questionnaire semi-structuré et administré, saisies avec CSPro 7.5 et analysées avec SPSS version 26. L'analyse multivariée a permis de déterminer les facteurs associés aux maladies bucco-dentaires avec leurs odds ratio et valeur p. Le seuil de significativité était de 0,05.

**Résultats:**

les maladies les plus fréquentes étaient la carie dentaire et la gingivite avec respectivement 80,4% et 64,8%. Les facteurs associés à la carie dentaire étaient l'âge de 12 à 25 ans (RC ajusté=1,4,8; p=0,001), les connaissances insuffisantes (RC ajusté=3,5; p=0,034) et les pratiques inadéquates (RC ajusté=1,8; p=0,013); ceux associés à la gingivite étaient le niveau d'instruction primaire (RC ajusté=5,2; p=0,04), les attitudes approximatives (RC ajusté=2,2; p=0,01 4) et les pratiques néfastes (RC ajusté=1 ,9; p=0,02).

**Conclusion:**

la carie dentaire et la gingivite sont fréquents chez les Pygmées Baka. Il est nécessaire de renforcer leur éducation et d'améliorer leur accessibilité aux soins bucco-dentaires.

## Abstract (en)

**Introduction:**

the epidemiological profile of oral diseases is characterised by disparities between specific groups. The purpose of this study was to assess oral health status of Baka Pygmies in Dimako, Cameroon.

**Methods:**

we conducted a cross-sectional study from January 1 to June 14, 2021 in the Baka camps in Dimako. A total of 205 individuals aged 12 years and older were recruited using non-probabilistic, consecutive sampling. Data were collected using a semi-structured, administered questionnaire then entered with CSPro 7.5, and analyzed with SPSS version 26. Multivariate analysis was used to identify factors associated with oral diseases, using their Odds ratios and p-values. The significance threshold was set at 0.05.

**Results:**

the most common diseases were dental caries and gingivitis, with prevalence rates of 80.4% and 64.8%, respectively. Factors associated with dental caries included age 12 to 25 years (adjusted OR=1.48; p=0.001), insufficient knowledge (adjusted OR=3.5; p=0.034), and inadequate practices (adjusted OR=1.8; p=0.013). Factors associated with gingivitis were primary education level (adjusted OR=5.2; p=0.04), approximate attitudes (adjusted OR=2.2; p=0.014) and harmful practices (adjusted OR=1.9; p=0.02).

**Conclusion:**

dental caries and gingivitis are prevalent among the Baka Pygmies. It is necessary to strengthen their education and improve their access to oral healthcare.

## Introduction

Les maladies bucco-dentaires constituent un problème de santé publique dans le monde et restent dominées par la carie dentaire, les maladies parodontales. Elles touchent près de 3,5 milliards de personnes avec environ 75% vivant dans des pays à faible revenu [1]. En effet, dans les pays développés, la carie dentaire touche 60% à 90% des enfants et 90% à 92% des adultes; les parodontites quant à elle affectent 5% à 20% de la population mondiale [2]. La région africaine a connu la plus forte augmentation du nombre de cas au cours des trois dernières décennies avec environ 480 millions de personnes touchées par ces maladies, représentant 43,7% de la population [3]. Au Sénégal, la prévalence de la carie dentaire a été estimée à 51% et celle de la maladie parodontale à 37% chez les enfants scolarisés [4]. Au Cameroun, des prévalences respectives de 29,9%, 39,2% pour la carie dentaire et les maladies parodontales ont été rapportées [3]. La morbidité croissante de ces maladies peut s'expliquer par la croissance démographique mais également la consommation croissante d'aliments sucrés, la consommation excessive d'alcool, l'utilisation du tabac, l'adoption des mauvaises habitudes d'hygiène bucco-dentaire ainsi qu'une faible sensibilisation [5,6].

Le profil épidémiologique des maladies bucco-dentaires n'est pas homogène au sein de la population. Il existe des disparités entre les groupes spécifiques parmi lesquels les peuples autochtones en l'occurrence les Pygmées [5,6]. Les études menées chez les Pygmées Baka dans la région du Sud ont rapporté des prévalences de 66,9% à 77,5% pour la carie dentaire et 60,7% pour la maladie parodontale [7,8]. Cette morbidité reste élevée par rapport à celle de la population générale. En effet, ces peuples de la forêt font face à une déforestation grandissante entrainant une modification de leur habitat naturel; l'urbanisation qui en résulte aboutit à un développement socio-économique avec pour corollaire la modification de leurs habitudes alimentaires au profit d'un régime riche en sucre raffiné et pauvre en minéraux et vitamines aggravant la morbidité bucco-dentaire chez ce peuple [9]. Par ailleurs, leur accès aux soins de santé bucco-dentaire reste limité par l'insuffisance des services de santé bucco-dentaire, le coût élevé de la prise en charge de ces maladies et l'utilisation de la pharmacopée traditionnelle [5].

Les Pygmées Baka font partir des groupes autochtones du Cameroun et sont retrouvés dans les régions administratives du Sud et de l'Est du pays [7,8]. Cependant, peu d'informations sont disponibles sur leur morbidité bucco-dentaire dans la région de l'Est ne permettant pas d'établir le profil épidémiologique des maladies bucco-dentaires de tous les Pygmées Baka. La connaissance de leurs besoins permettra d'élaborer des interventions adaptées à leurs réalités culturelles et de les intégrer dans la politique nationale de santé bucco-dentaire du pays; en outre, leur implémentation sera adossée aux objectifs de la stratégie régionale africaine pour la santé bucco-dentaire 2016-2025 [5]. Cette stratégie fournit aux États membres des orientations permettant d'intégrer les affections bucco-dentaires dans la lutte contre les maladies non transmissibles dans le cadre de la couverture sanitaire universelle et dont l'un de ses objectifs est de renforcer la capacité du système de santé à assurer une prévention et une maîtrise intégrées des maladies bucco-dentaires. Ainsi, quel est le profil des maladies bucco-dentaires chez les Pygmées Baka dans la Région de l'Est au Cameroun? Quels sont les facteurs associés à ces maladies? C'est dans ce contexte que nous nous proposons d'identifier les maladies bucco-dentaires chez les Pygmées Baka de l'Est et de déterminer les facteurs associés à ces maladies.

## Méthodes

**Type d'étude:** nous avons mené une étude transversale au sein des campements des Pygmées Baka dans la région de l'Est du Cameroun.

**Cadre et période de l'étude:** l'étude s'est déroulée entre le 1^er^ janvier et le 14 juin 2021 dans l'arrondissement de Dimako situé dans la région administrative de l'Est Cameroun. En effet, Dimako est la ville dans laquelle sont retrouvés les campements Baka. Ces derniers occupent les villages Lossou, Nkoumadjap, Mayos et Nkolbikon, situés à des kilomètres du centre-ville.

**Population de l'étude:** notre population cible était l'ensemble des habitants des groupements Pygmées Baka de Dimako. Etait inclus tout individu résidant et appartenant aux campements des pygmées Baka âgé au moins de 12 ans et ayant donné son consentement éclairé écrit ou verbal et avec l'assentiment parental pour les participants de 12 à 20 ans. Nous avons exclu tout individu ayant interrompu l'entretien pour quelque raison que ce soit. Nous avons réalisé un recrutement consécutif et exhaustif. La taille minimale de l'échantillon a été obtenue en utilisant la formule ci-après:


$$N=\left(Z_{1-\alpha /2}{\sqrt{\left(P\left(1-P\right)/d\right)}}^{2}\right)$$

avec l'abscisse de la courbe de la loi normale correspondant au niveau de confiance de 95% Z_1-&/2_=1.96, une marge d'erreur α=5%, une précision d=5% et une prévalence de la carie dentaire de 59,16% dans une population rurale au Bénin en 2016 [9]. Ensuite, elle a été ajustée à 20% afin de prendre en considération les potentiels retraits et refus. Ainsi, une taille minimale de 182 individus a été obtenue.

**Collecte des données**: les données ont été recueillies à l'aide d'un questionnaire semi-structuré administré par une équipe de huit enquêteurs préalablement briefés à l'outil pendant deux jours par les investigateurs.

**Caractéristiques socio-démographiques**: la première section portait sur les caractéristiques socio-démographiques; les variables collectées étaient l'âge (en années) rapporté en utilisant tout document officiel (acte de naissance, livret scolaire) ou une méthode empirique reposant sur le cycle annuel de reproduction des arbres fruitiers avec l'aide des anciens des clans (réservée pour tous ceux ne disposant pas de document officiel, estimée à 10%) le sexe (masculin ou féminin), la profession (élève/étudiant, cultivateur, employé) et le niveau scolaire reparti en, primaire, secondaire, universitaire.

**Connaissances, attitudes et pratiques**: dans la seconde section relative à l'évaluation des connaissances des Pygmées Baka sur les maladies bucco-dentaires, les questions ont porté sur leur existence, leurs manifestations, leur prise en charge et les méthodes de prévention [10,11]. La troisième section était consacrée à l'identification les attitudes des participants vis-à-vis des maladies bucco-dentaires [10,11]. Quant à la quatrième section, elle a permis de déterminer leurs pratiques en cas de maladie.

**Examen bucco-dentaire:** la cinquième section a porté sur l'examen buccal; il a été réalisé à l'aide d'un miroir buccal, d'un abaisse-langue, d'une sonde dentaire N°17, d'une sonde parodontale de l'OMS graduée de 3,5 à 5,5 mm avec une extrémité en forme de boule de 0,5 mm de diamètre ainsi qu'une torche frontale [9,12]. La nature et les caractéristiques cliniques des lésions bucco-dentaires ont été recueillies. A cet effet, une dent était considérée cariée lorsqu'elle présentait un puits/fissure/cavité évidente et détectable au passage de la sonde sur l'une des surfaces dentaires ou un émail déminéralisé [13]. Une dent avec une obturation temporaire/scellée cariée était incluse; toute dent était considérée obturée lorsqu'elle présentait une ou plusieurs restaurations permanentes à cause de la carie; toute dent perdue des suites d'une carie était considérée absente [13]. En outre, l'indice CAOD (C=dent cariée; A= dent absente; O= dent obturée) a été évalué en faisant la moyenne de la somme du nombre des dents permanentes cariées, absentes et obturées. La maladie parodontale a été définie avec la présence d'au moins un des signes/symptômes suivants: saignement au brossage, gencive enflammée/enflée/rétractée quelle que soit la cause, mobilité des dents permanentes; la classification des maladies parodontales s'est faite en utilisant les critères de Chicago 2017 [13,14]. Quant à la perte de substance dentaire minéralisée, elle était qualifiée d'abrasion lorsqu'elle a été provoquée par un objet externe, d'attrition par le contact entre les dents, d'usure par un processus physique non carieux [1]. Les ulcérations étaient dites non spécifiques lorsqu'elles étaient survenues en dehors de tout contexte fébrile au cours des deux mois précédant l'enquête et/ou en absence de toute maladie systémique rapportée par les participants [1]. L'hygiène bucco-dentaire a été appréciée à l'aide de l'indice de plaque de Silness et Loe. Cet indice comportait quatre degrés (0=absence de plaque, 1=film adhérant au bord marginal, 2=accumulation modéré visible à l'œil nu, 3=plaque abondante) en fonction de la quantité de plaque ou de tartre sur la couronne dentaire et la gencive marginale; le degré le plus élevé retrouvé était celui de toute la cavité orale. Ces degrés correspondaient respectivement aux niveaux d'hygiène excellent, bon, moyen et modéré [12]. Après cet examen, les personnes présentant des affections bucco-dentaires ont été informés sur leur état de santé bucco-dentaire et un plan de traitement leur a été proposé. Une sensibilisation au cas par cas a été faite pour les informer sur la nécessité d'un suivi.

**Considérations éthiques**: la clairance éthique a été obtenue auprès du comité d'Ethique de la Faculté de Médecine et des Sciences Biomédicales de l'Université de Yaoundé I par l' autorisation N°166/UY1/FMSB/VDRC/DAASR/CSD. Une autorisation administrative N°264/L/MINSANTE/SG/DRSPE/BFP a été délivrée par le Délégué Régional de la Santé Publique de l'Est. Le consentement éclairé écrit préalablement traduit en langue locale pour les participants ne s'exprimant pas en français a été obtenu au préalable et les personnes âgées de 12 à 20 ans ont donné leur assentiment et leurs parents, leur consentement éclairé et informé. Cette étude a été réalisée conformément à la déclaration d'Helsinki. Toutes les informations recueillies auprès des participants sont restées confidentielles. Les personnes présentant des pathologies bucco-dentaires ont été informés de leur état de santé bucco-dentaire et se sont vu proposer un plan de traitement.

**Analyses statistiques**: les données collectées ont été saisies avec CSPro 7.5 et analysées avec SPSS version 26. Les variables qualitatives ont été présentées avec leurs effectifs et pourcentages. Le test de Kolmogrov-Smirnov a été utilisé pour évaluer la normalité des variables quantitatives; celles ayant une distribution normale ont été résumées avec leur moyennes et écart type tandis que la médiane et l'intervalle interquartile étaient utilisées pour celles ayant une distribution non normale. Ensuite, nous avons catégorisé les niveaux de connaissances, attitudes et pratiques. Pour chacune des questions de ces composantes, chaque réponse juste valait un point et un score en pourcentage a été calculé afin d'obtenir les catégories en se basant sur les travaux de Essi *et al*. (Tableau 1) [8]. Afin d'analyser la relation entre les maladies bucco-dentaires et les autres variables, nous avons réalisé une analyse bivariée entre les principales maladies, variables dépendantes et les caractéristiques démographiques, les niveaux de connaissances, d'attitudes et pratiques. Les variables présentant une valeur p significative ou proche ont ensuite été inclues dans le modèle de régression logistique binaire. Ainsi, l'association recherchée entre les maladies bucco-dentaires et les variables retenues a été exprimée au moyen des rapports de côte assortis des leurs intervalles de confiance à 95%; les tests statistiques de Chi^2^ et de Fisher pour les effectifs inférieur à cinq ont été utilisés avec un seuil de significativité de 0,05.

**Tableau 1:** grille de cotation des connaissances, attitudes et pratiques en santé bucco-dentaire utilisée chez les Pygmées Baka à Dimako au Cameroun, 2021

Composantes	Score (%)	Interprétations
**Connaissances**	Moins de 25	Mauvaises
	25 à 50	Insuffisantes
	50 à 70	Moyennes
	Plus de 70	Bonnes
**Attitudes**	Moins de 25	Néfastes
	25 à 50	Erronées
	50 à 70	Approximatives
	Plus de 70	Justes
**Pratiques**	Moins de 25	Néfastes
	25 à 70	Inadéquates
	Plus de 70	Adéquates

## Résultats

**Description de la population d'étude:** pendant la période d'étude, 300 individus ont bénéficié d'un examen bucco-dentaire et 205 remplissant nos critères d'inclusion ont été retenus.

### Caractéristiques socio-démographiques des participants

Nous avons retrouvé une prédominance masculine avec un sex-ratio de 1,44: 1. Vingt-sept (13,2%) n'avaient jamais été scolarisés et 175 (83,3%) étaient des agriculteurs. L'âge moyen était de 25,41 (±9,3) ans; 120 (58,5%) participants avaient un âge compris entre 12 et 25 ans (Tableau 2).

**Tableau 2:** caractéristiques socio-démographiques des Pygmées Baka de Dimako au Cameroun, 2021

Caractéristiques socio-démographiques	N=205 n (%)
**Genre**	
Masculin	121 (59)
Féminin	84 (41)
**Niveau scolaire**	
Aucun	27 (13,2)
Primaire	146 (71,2)
Secondaire	31 (15,1)
Supérieur	1 (0,5)
**Profession**	
Agriculteur	175 (83,3)
Elève/étudiant	25 (12.1)
Employé	5 (2,4)
**Age (en années)^2^**	
12-25	120 (58,5)
26-38	64 (31,2)
≥ 39	21 (10,2)

^2^f± écart type: 25,41 ± 9,3 ans

### Connaissances, d'attitudes et de pratiques des Pygmées Baka sur les maladies bucco-dentaires

L'évaluation du niveau des connaissances sur les maladies bucco-dentaires a montré qu'il était bon chez 121 (59%) participants; 65 (31,7%) avaient des attitudes approximatives. L'identification des pratiques a montré que 124 (60,5%) avaient un niveau de pratique inadéquat (Tableau 3).

**Tableau 3:** connaissances, attitudes et pratiques en santé bucco-dentaire des Pygmées Baka de Dimako au Cameroun, 2021

Composantes évaluées	N = 205 n (%)
**Connaissances**	
Mauvaises	50 (24,4)
Insuffisantes	3 (1,5)
Moyennes	31 (15,1)
Bonnes	121 (59)
**Attitudes**	
Néfastes	46 (22,4)
Erronées	55 (26,8)
Approximatives	65 (31,7)
Justes	40 (19,1)
**Pratiques**	
Néfastes	33 (16,1)
Inadéquates	124 (60,5)
Adéquates	48 (23,4)

### Etat bucco-dentaire des Pygmées Baka

**Figure 1:**
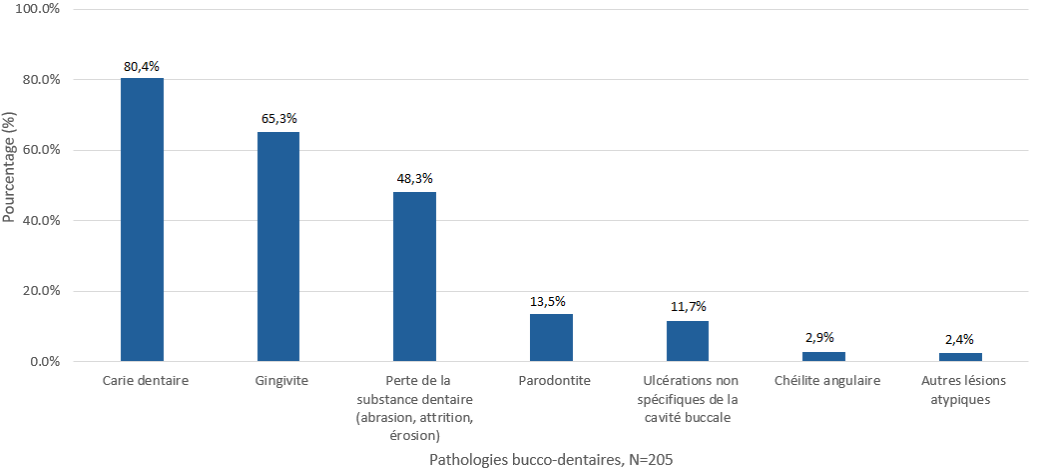
pathologies bucco-dentaires retrouvées chez les Pygmées Baka de Dimako au Cameroun, 2021

L'examen bucco-dentaire a révélé que la prévalence de la carie dentaire était de 80,4% (IC à 95%:60,3 à 87,5); celles de la gingivite et de la parodontite étaient respectivement de 64,8% (IC à 95%:56,3 à 71,8) et 13,5% (IC à 95 %:10,2 à 15,6). Les pathologies retrouvées sont présentées dans la Figure 1. L'évaluation du niveau de plaque a révélé que 30 (14,6%) participants avaient une bonne hygiène bucco-dentaire et 105 (51,2%) ont déclaré ne pas se brosser les dents. L'indice moyen CAOD (dents cariées, absentes ou obturées) de notre population d'étude était de 3,02 (Tableau 4).

**Tableau 4:** état bucco-dentaire des Pygmées Baka de Dimako au Cameroun, 2021

Etat bucco-dentaire	N=205 n (%)
**Indice de Loe et Sillness (Hygiène bucco-dentaire)2**	
Degré 0 (Excellente)	30 (14,6)
Degré 1 (Bonne)	43 (20,9)
Degré 2 (Modérée)	117 (57)
Degré 3 (Mauvaise)	15 (7,3)
**Fréquence de brossage (nombre/jour)**	
Pas de brossage	105 (51,2)
Une fois	27 (13,1)
Deux fois	21 (10,2)
Trois fois	10 (4,8)
**Etat de la denture**	
Indice CAOD^3^	3,09
Dents cariées	465
Dents absentes	165
Dents obturées	5

2 Degré 0: pas de plaque ou de tartre, Degré 1: présence d´une couche fine de tartre, Degré 2: d épote de plaque visible à l´œil nu, Degré 3: accumulation de plaque importante; 3 Moyenne; CAOD: dents cariées, absentes, obturées.

### Facteurs associés à l'état bucco-dentaire chez les Pygmées Baka

***Facteurs associés à la carie dentaire chez les Pygmées Baka:*** les facteurs statistiquement associés à la carie dentaire chez les Pygmées étaient l'âge compris entre 12 et 25 ans (RC ajusté=1,4,8; p=0,001), les connaissances insuffisantes (RC ajusté=3,5; p=0,034) et les pratiques inadéquates (RC ajusté=1,8; p=0,013) (Tableau 5).

**Tableau 5:** facteurs associés à la carie dentaire chez des Pygmées Baka de Dimako au Cameroun, 2021

Variables	Carie dentaire		Rapport de côte brut (IC à 95%^2)^	Rapport de côte ajusté (IC à 95%^2^)	Valeur p ajustée^3^
	Oui, N=165^1^	Non, N=40^1^			
**Genre**					
Féminin	66 (78,6)	18 (21,4)	1	**-**	**-**
Masculin	99 (81,8)	22 (18,2)	1,22 (0,2-3,31)	**-**	**-**
**Age (en années)**					
≥ 39	20 (92,6)	1 (7,4)	1	1	-
12-25	101 (84,2)	19 (15,8)	0,26 (0,01- 0,9)	1,4 (1,01-4,2)	0,001 *
26-38	44 (68,7)	20 (31,3)	0,11 (0,06-0,75)	0,5(0,01-3,9)	0,1
**Niveau scolaire**					
Aucun	25 (92,6)	2 (7,4)	1	-	-
Primaire	117 (84,2)	29 (15,8)	0,32 (0,07-4,51)	-	0,50
Secondaire	22 (71)	9 (29)	0,19 (0,013-3,51)	-	0,96
**Profession**					
Elève/Etudiant	10 (40)	15 (60)	1	-	-
Agriculteurs	153 (87,4)	22 (12,6)	10,43 (0,6-18,09)	-	-
Employés	2 (40)	3 (60)	1 (0,1-4,6)	-	-
**Connaissances**					
Bonnes	95 (78,5)	26 (21,5)	1	1	-
Mauvaises	44 (88)	6 (22)	2 (0,8-5,8)	3,5 (1,1-7,8)	0,034*
Insuffisantes	1 (33,3)	2 (75)	0,13 (0,03-0,9)	4,03 (0,13-7,8)	0,14
Moyennes	25 (80,6)	6 (19,4)	1,14 (0,11-4,43)	0,5(0,02-3,4)	0,41
**Attitudes**					
Justes	30 (75)	10 (25)	1	1	-
Néfastes	31 (67,4)	15 (32,6)	0,68 (0,02-0,93)	2,6 (0,07-3,99)	0,3
Erronées	45 (81,8)	9 (18,2)	1,66 (0,01-4,8)	1,23(0,02-4,8)	0,4
Approximatives	59 (90,8)	6 (9,2)	3,3 (0,16-6,2)	0,15 (0,02-4,5)	0,44
**Pratiques**					
Adéquates	42 (87,5)	6 (12,5)	1	1	-
Néfastes	24 (72,7)	9 (27,3)	0,38 (0,05-0,92)	0,45 (0,03-2,9)	0,1
Inadéquates	99 (79,8)	25 (20,2)	0,56 (0,019-0,7)	1,8 (1,09-5,9)	0,013 *

**^1^**n (%); IC à 95%**^2^**: Intervalle de Confiance à 95 %; **^3^**Tests de Chi^2^ (n>5) et exact de Fisher (n < 5); *: valeur p ajustée significative (< 0,05).

***Facteurs associés à la gingivite chez les Pygmées Baka*:** l'analyse a rapporté que les variables statistiquement associées à la gingivite étaient les le niveau primaire d'instruction (RC ajusté=5,2; p=0,04), les attitudes approximatives (RC ajusté =2,2; p=0,014) et les pratiques néfastes (RC ajusté =1,9; p=0,02) (Tableau 6).

**Tableau 6:** facteurs associés à la gingivite chez les Pygmées Baka de Dimako au Cameroun, 2021

Variables	Gingivite		Rapport de côte (IC À 95%^2^)	Rapport de côte ajusté (IC À 95%^2^)	Valeur p ajustée 3
	Oui, N=134^1^	Non, N=71^1^			
**Genre**					
Féminin	76 (62,8)	45 (37,2)	1	**-**	**-**
Masculin	58 (69)	26 (31)	1,32 (0,52-5,7)	-	**-**
**Age (en années)**					-
12-25	84 (78,3)	36 (21,7)	1	-	**-**
26-38	36 (87,5)	28 (12,5)	0,55 (0,07-5,6)	-	-
≥ 39	14 (85,7)	7 (14,3)	0,85 (0,04-4,8)	-	**-**
**Niveaux d’instruction**					
Aucun	4 (14,8)	23 (85,2)	1	1	-
Primaire	108 (74)	38 (26)	16,34 (7,8-25,2)	5,2 (1,05-9,7)	0,04*
Secondaire	21 (67,7)	10 (32,3)	12,1 (5,6-21,4)	2,5 (0,04-5,8)	0,9
**Profession**					
Elèves/Etudiants	10 (40)	15 (60)	1	**-**	**-**
Agriculteurs	121 (69,1)	54 (30,9)	3,4 (0,01-6,04)	-	**-**
Employés	3 (60)	2 (40)	2,25 (0,04-5,15)	**-**	**-**
**Connaissances**					
Mauvaises	35 (70)	15 (30)	1	1	-
Insuffisantes	2 (66,7)	1 (33,3)	0,85 (0,01-0,93)	1,75 (0,02-4,8)	0,11
Moyennes	21 (67,7)	10 (32,3)	0,9 (0,06-4,5)	0,7 (0,01-3,3)	0,7
Bonnes	76 (62,8)	45 (37,2)	0,72 (0,12-2,58)	0,2 (0,06-3,8)	0,96
**Attitudes**					
Justes	28 (70)	12 (30)	1	1	-
Néfastes	26 (56,5)	20 (43,5)	0,55 (0,02-0,85)	1,5 (0,09-3,5)	0,63
Erronées	30 (54,5)	25 (45,5)	0,51 (0,18-4,17)	1,1 (0,05-2,9)	0,33
Approximatives	50 (77)	14 (33)	1,5(1,02-5,24)	2,2 (1,01-4,2)	0,014*
**Pratiques**					
Adéquates	36 (75)	12 (25)	1	1	-
Néfastes	29 (87,9)	4 (12,1)	2,41 (1,09-5,7)	1,9 (1,04-4,6)	0,02*
Inadéquates	69 (55,6)	55 (44,4)	0,42 (0,02-0,77)	2,2 (0,09-5,1)	0,6

**^1^**n (%); IC à 95 %**^2^**: Intervalle de Confiance à 95 %; **^3^**Tests de Chi^2^ (n>5) et exact de Fisher (n < 5); *: valeur p ajustée significative (< 0,05).

## Discussion

L'examen bucco-dentaire a révélé que les maladies prédominantes étaient la carie dentaire et la gingivite avec respectivement 80,4% et 64,8% des cas. Ces résultats restent inférieurs à ceux retrouvés par Pagou *et al*. en 2017 qui ont retrouvé respectivement 96,8% et 74,7% dans un groupe spécifique à l'Extrême-nord du Cameroun [15]. Cependant, ils restent supérieurs à ceux de Okoko *et al*. au Congo en 2013 qui avaient trouvé 53% pour la carie dentaire ainsi que de Aboubakar *et al*. au Cameroun en 2017 avec 50% de participants présentant les lésions gingivales [16,17]. Cette prévalence élevée de la carie pourrait se justifier par l'augmentation accrue de la consommation des aliments riches en sucres raffinés [18,19]. L'urbanisation a entrainé une modification du biotope de ce peuple avec pour conséquence une modification de leurs habitudes alimentaires traditionnelles les emmenant à consommer des aliments sucrés comme les confiseries, le chocolat, la pâtisserie, les boissons sucrées dont la dégradation conduit à la production des acides qui vont causer la carie. La prédominance des gingivites parmi les maladies parodontales quant à elle s'expliquerait par le non-respect des règles d'hygiène bucco-dentaire favorisant le dépôt de tarte, support pour la flore bactérienne à l'origine de l'inflammation de la gencive, point de départ de la maladie parodontale; en effet, 57% des individus interrogés avaient un dépôt de plaque visible à l'œil nu et la moitié déclarait ne pas appliquer ces règles. Environ 60% des participants avaient un âge compris 12 et 25 ans avec 71,2% ayant atteint le cycle primaire d'études ce qui pourrait expliquer une insuffisance de l'appropriation des règles d'hygiène bucco-dentaire; en outre, le caractère nomade de ce peuple influencerait l'incorporation de ces règles dans leurs habitudes en limitant leur suivi [7,8].

Nous avons trouvé que les Pygmées ayant des connaissances mauvaises avaient 4,03 fois plus de risque d'avoir la carie dentaire (p=0,034) par rapport à ceux ayant des connaissances bonnes. Bien que 89,9% des participants connaissaient l'existence des maladies bucco-dentaires, 34% ne reconnaissaient pas la cavitation comme signe de carie dentaire. Ces résultats se rapprochent de ceux de Garé *et al*. au Burkina Faso en 2019 qui ont retrouvé 40,2% [20]. Ceci traduit une certaine méconnaissance de la pathologie carieuse. Près de la moitié des participants, soit 52,2% ne savaient pas que la sensibilité dentaire était un signe de maladie. Ces résultats corroborent ceux obtenus par Garé *et al*. en 2019 qui mentionnaient 55,3% [20]. En effet, les sensibilités sont souvent les premiers signes et symptômes d'une maladie bucco-dentaire sous-jacente mais restent négligées ou ignorées, ce qui conduit aux complications [7,8]. Environ 13,7% ont cité la carie dentaire comme définition de maladie de la dent. Cependant, de Amorim *et al*. ont obtenu des résultats légèrement supérieurs avec 29% [21]. Concernant les connaissances sur les moyens de prévention de la carie, 22,3% avaient identifié le brossage comme moyen pour éviter la carie dentaire. Ces résultats sont inférieurs à ceux de Garé *et al*. au Burkina en 2019 qui retrouvaient 33,7% dans son étude menée en zone semi-urbaine [20]. Nos résultats pourraient s'expliquer par l'intensification des campagnes de promotion de la santé bucco-dentaire organisées par les associations locales et internationales à l'endroit de ces peuples au cours de ces dernières années. Par ailleurs, l'hémorragie gingivale n'a pas été identifié comme signe de maladie par 64%. Similairement, une étude menée par Mengue chez les Pygmées Baka au Sud du Cameroun [7] en 2020 qui retrouvait 73,5%. Ces résultats pourraient s'expliquer par le fait que les participants avaient des connaissances limitées sur les manifestations des maladies bucco-dentaires. Concernant la prévention des gingivites, nous avons retrouvé que 34,6% des participants connaissaient que l'utilisation de la brosse à dent ou de la tige traditionnelle était un moyen de prévention; Afoumpam *et al*. chez les enfants des Pygmées Baka au Sud du Cameroun en 2017 avaient obtenu des résultats similaires avec 33,6% [8]. Ces résultats pourraient s'expliquer par le fait que les participants n'adoptaient pas de bonnes habitudes en matière d'hygiène. Par ailleurs, 92,8% ont affirmé n'avoir jamais eu recours à un spécialiste; ceci pourrait se justifier par l'ignorance, le coût élevé des soins, l'éloignement des structures hospitalières [16,20-24]. Concernant la réaction face aux maladies bucco-dentaires, 78% ont affirmé avoir recours au traitement traditionnel. Nos résultats sont supérieurs à ceux retrouvés par Kaboré *et al*. en 2016 au Burkina Faso qui avaient trouvé 10,5% [24]. Toutefois, ils se rapprochent de ces deux études menées chez les Pygmées Baka au Sud du Cameroun qui ont retrouvé 85 et 91% [7,8]. Ces résultats suggèrent que ces peuples restent rattachés à leur pharmacopée traditionnelle reposant sur l'utilisation des produits faits à base de plantes et d'écorces d'arbres qui auraient des vertus antalgiques et antibiotiques mais dont le conditionnement, la posologie et les indications restent à encadrer pour une meilleure exploitation [7,8]. Les participants avec des attitudes approximatives avaient significativement 2,2 fois plus de risque d'avoir la gingivite par rapport à ceux ayant des attitudes justes (p=0,014). En effet, nous avons retrouvé que 79,1% n'utilisaient pas la brosse à dent. Similairement Mengue *et al*. 2020 au Cameroun avaient retrouvé 83,4% [7]. Ceci traduit un manque de connaissances. Dans cette étude, les pratiques inadéquates (RC ajusté=1,8; p=0,013) et néfastes (RC ajusté=2,2; p=0,02) étaient respectivement associées à la présence de carie dentaire et de gingivite respectivement. Les pratiques néfastes et inadéquates identifiées chez les participants s'expliqueraient par les croyances telles que présentées par la théorie du comportement planifié; elle suggère que ces croyances influenceraient indirectement les comportements liés à la santé, en affectant la gravité perçue, la vulnérabilité, les avantages et les obstacles perçus [25-27].

### Limites

Plusieurs études ont porté sur les groupes spécifiques au Cameroun et quelques-unes sur les Pygmées Baka spécifiquement. Comparativement à ces études, ce travail faisait partir des enquêtes pilotes menées chez les Pygmées Baka à l'Est du pays. Il a permis également de disposer de données récentes pouvant servir à la prise de décision en contribuant à établir le profil épidémiologique des maladies bucco-dentaires chez l'ensemble les Pygmées Baka de notre pays ainsi que les facteurs associés. Cependant, quelques faiblesses ont été recensées notamment l'hésitation et la réticence de certains habitants des campements à participer à notre enquête; elles ont contribué à réduire le nombre de participants bien que nous ayons dépassé la taille minimale de notre échantillon.

## Conclusion

Les maladies bucco-dentaires les plus fréquentes chez les Pygmées Baka de Dimako sont la carie dentaire et la gingivite. La présence de ces maladies met en évidence un besoin identifié en connaissances, attitudes et pratiques. En effet, la politique de santé devrait intégrer l'éducation bucco-dentaire de ce peuple ainsi que l'accessibilité aux soins de santé bucco-dentaire afin de limiter leur morbidité ainsi que leurs conséquences.

### Etat des connaissances sur le sujet

- La charge épidémiologique des maladies bucco-dentaires est marquée par des inégalités affectant également les groupes spécifiques;
- Les maladies bucco-dentaires sont dominées par la carie, la gingivite, les édentations et les cancers de la bouche.

### Contribution de notre étude à la connaissance

- La morbidité bucco-dentaire est plus importante chez les Pays Baka à Dimako comparativement à la population générale;
- Il existe un besoin en éducation bucco-dentaire afin d'améliorer leurs connaissances, leurs attitudes et pratiques.
